# The Applicability of ADA, AFU, and LAC in the Early Diagnosis and Disease Risk Assessment of Hepatitis B-Associated Liver Cirrhosis and Hepatocellular Carcinoma

**DOI:** 10.3389/fmed.2021.740029

**Published:** 2021-09-07

**Authors:** Wei Zhang, Zhong Chen, Chengjun Xue, Yao Zhang, Lipei Wu, Jichao Zhu, Shihai Xuan, Jiale Tian, Zhi Pang

**Affiliations:** ^1^Department of Laboratory Medicine, Jiaozuo Fifth People's Hospital, Jiaozuo, China; ^2^Department of Laboratory Medicine, Luoyang Hospital of Traditional Chinese Medicine, The Affiliated Hospital of Henan University of Chinese Medicine, Luoyang, China; ^3^Department of Gastroenterology, Jianhu Hospital of Nantong University, Yancheng, China; ^4^Department of Laboratory Medicine, Dongtai People's Hospital & Dongtai Hospital of Nantong University, Yancheng, China; ^5^Department of Laboratory Medicine, Huzhou Central Hospital, Affiliated Central Hospital of Huzhou Normal University, Huzhou, China; ^6^Department of Laboratory Medicine, Tongji Hospital, Tongji University School of Medcine, Shanghai, China; ^7^Department of Gastroenterology, The North District of the Affiliated Suzhou Hospital of Nanjing Medical University, Suzhou, China

**Keywords:** ADA, AFU, LAC, hepatitis, liver cirrhosis, hepatocellular carcinoma, diagnostic value, risk assessment

## Abstract

**Objective:** This study aimed to evaluate the applicability of adenosine deaminase (ADA), α-l-fucosidase (AFU), lactic acid (LAC), and their combined detection in the early diagnosis of chronic hepatitis B (CHB), liver cirrhosis (LC), and hepatocellular carcinoma (HCC).

**Methods:** A retrospective analysis of hepatitis B-positive liver disease patients admitted between 2015 and 2020 was conducted. The receiver operating characteristic (ROC) curve was used to determine the diagnostic value of each indicator in LC and HCC, and binary logistic regression analysis was performed to determine the factors and risks related to the occurrence of the two conditions.

**Results:** The levels of ADA, AFU, and LAC were significantly increased in patients with CHB, LC, and HCC (*p* < 0.05). The ROC curve showed that the sensitivity and specificity of ADA, AFU, LAC, and their combined detection in the CHB and LC groups as well as in the LC and HCC groups reflected different degrees of clinical value. In the CHB and LC groups, the adjusted odds ratio (OR) values of ADA, AFU, and LAC among patients in the high-level group were 3.218, 1.859, and 11.474, respectively, when the median was considered the cutoff point. When quartiles were considered the cutoff point, the OR risk values of the adjusted levels of ADA, AFU, and LAC were higher than those of the lowest-level group (Q1) (*p* < 0.05). In the LC and HCC groups, the adjusted OR values of ADA, AFU, and LAC among patients in the high-level group were 0.967, 2.365, and 38.368, respectively. When quartiles were considered the cutoff point, the OR risk values of AFU and LAC levels were higher than those of the lowest-level group (Q1) (*p* < 0.05).

**Conclusion:** ADA, AFU, and LAC demonstrated good value in the early diagnosis of LC and HCC. The combined detection of ADA+AFU+LAC is more effective than single detection for the early diagnosis of the two conditions. ADA, AFU, and LAC can serve as risk predictors of LC, while AFU and LAC can be considered early risk predictors of HCC.

## Introduction

Hepatitis B virus (HBV) infection is a global public health problem, and the number of hepatitis B surface antigen (HBsAg) carriers is ~250 million (1). Chronic hepatitis B (CHB) can cause gradual aggravation of liver injury, and without intervention, ~40% of the patients with the infection develop liver cirrhosis (LC). In addition, ~30% of the patients with LC develop hepatocellular carcinoma (HCC) after 10 years (2). LC was previously considered irreversible; however, it is now known that the condition can be reversed through oral anti-nucleotide drug therapy (3). Liver cancer is a common malignancy of the organ and the fourth most common cause of cancer-related deaths (4). Some liver cancers can progress insidiously in patients with normal liver function, and early diagnosis may not be possible due to non-specific symptoms (5). Therefore, early and accurate diagnosis of LC and liver cancer is important for the choice of appropriate treatment programs. Liver biopsy is the gold standard technique for the assessment of LC and cancer. However, considering its invasiveness, complexity, and potential risks, liver biopsy cannot be performed routinely in most patients (6). Transient elastography is a superior tool to diagnose liver fibrosis; however, it tends to be affected by several factors such as diet, obesity, ascites, and rib gap width (7). The common clinical indicators, alpha-fetoprotein level and liver function, are not ideal for the early diagnosis and prognostication of the conditions. To date, there are no effective markers to predict the progression of chronic liver disease (8).

Adenosine deaminase (ADA), as a key enzyme in purine nucleoside and DNA metabolism, plays an important role in the maintenance and development of the human immune, nervous, and vascular systems (9). Studies have reported higher serum ADA levels in patients with esophageal, gastric, breast, and ovarian cancers than in the healthy population (10). Lactic acid (LAC) is a metabolite of glycolysis produced in the bones, muscles, brain, and red blood cells. The liver is responsible for the clearance of 70% of LAC in humans, and liver damage can cause mitochondrial oxidation, leading to increased LAC levels (11). The lysosomal enzyme, α-l-fucosidase (AFU), is widely present in tissues and body fluids. A study reported a significant increase in AFU levels in patients with liver cancer compared to those with benign liver disease (12). In this study, we aimed to evaluate the applicability of ADA, AFU, LAC, and their combined detection in the early diagnosis of hepatitis B-associated LC and HCC. In addition, we aimed to determine the best cutoff value of the aforementioned markers for LC and early HCC and to provide a reference for the delay in the occurrence as well as for the early diagnosis and timely treatment of the two conditions to improve the quality of life and prolong the survival of patients.

## Materials and Methods

### Patients

We conducted a retrospective analysis of hepatitis B-positive liver disease patients admitted to Jiaozuo Fifth People's Hospital, Luoyang Traditional Chinese Medicine Hospital, Dongtai Hospital, affiliated to Nantong University, Yancheng Jianhu People's Hospital, Huzhou Central Hospital, and Shanghai Tongji Hospital from 2015 to 2020. We included 240 patients diagnosed with CHB (CHB group), 281 patients with LC (LC group), and 280 patients with early HCC (HCC group). The diagnoses of CHB and LC were based on the CHB prevention and treatment guidelines (13), and that of HCC was based on the liver cancer diagnosis guidelines (14). The inclusion criteria were as follows: HBsAg positive for more than 6 months, chronic HBV infection confirmed by histopathology, and early HCC (size of the lesion of 3 cm or less than three lesions). The exclusion criteria were patients with other types of liver disease, those who had received drugs that could cause liver damage within 6 months before admission, those with tumors in other parts of the body and/or hematological disease, those who had undergone organ transplantation, and patients with immune deficiencies.

### Clinical Information and Laboratory Examination

The following clinical and laboratory data of the included patients were recorded: age, sex, alpha-fetoprotein (AFP), carcinoembryonic antigen (CEA), aspartate aminotransferase (AST), alanine aminotransferase (ALT), total bilirubin (TBIL), direct bilirubin (DBIL), total protein (TP), albumin (ALB), alkaline phosphatase (ALP), gamma-glutamyltransferase (GGT), ADA, AFU, and LAC levels, among other markers.

### Statistical Analysis

SPSS 25.0 was used to perform the statistical analyses on the data that met the requirements, and normal test analyses were performed on the measurement data using the Kolmogorov–Smirnov test. Normally distributed data are presented as x ± standard deviation (SD). The measurement data of normal distribution between groups were compared using analysis of variance, and the count data were evaluated by the χ^2^-test. The measurement data of skewed distribution are presented as the median (M) and percentile (P25, P75). The measurement data of skewed distribution were compared by independent sampling using the Kruskal–Wallis H test. Pairwise comparisons were performed using the Bonferroni correction method for groups with differences in the overall test. GraphPad Prism software was used to construct the receiver operating characteristic (ROC) curve of each index and combined test to determine the sensitivity, specificity, optimal cutoff value, Youden index, negative predictive value (NPV), and positive predictive value (PPV) of each index in patients with LC and HCC. The area under the curve (AUC) was used to assess the accuracy of the tests. We performed binary logistic regression analysis to calculate the joint predictors of ADA, AFU, and LAC and the *Z*-test to compare the area under the ROC curve of each marker. The median (P50) and quartiles (P25, P50, and P75) were considered the cutoff points, and binary logistic regression analysis was performed to evaluate the risk of ADA, AFU, and LAC levels in LC and HCC. Factors with statistical significance in the univariate analysis (*p* < 0.10) were included in the multivariate logistic regression analysis, and binary logistic regression analysis was performed to calculate the single factor, multivariate-adjusted odds ratio, and 95% confidence interval (CI) values based on maximum likelihood estimation. The difference was considered statistically significant when *p*-value was < 0.05.

## Results

### Characteristics of the Enrolled Patients

The sex, age, AFP, AST, TBIL, ALP, and GGT levels of patients in the three groups were not statistically significant (*p* > 0.05). The ALB level of patients in the LC group was higher than that of patients in the CHB group, and the difference was statistically significant (*p* < 0.05). The CEA level was higher and the TP and ALB levels were lower among patients in the HCC group compared to those in the LC group, and the differences were statistically significant (*p* < 0.05). The TP, ALB, and DBIL levels were lower and the CEA and ALT levels were higher among patients in the HCC group compared to those in the CHB group, and the differences were statistically significant (*p* < 0.05; Table 1).

**Table 1:** Comparison of basic clinical data of the three groups of patients.

**Project**		**CHB group (*n* = 240)**	**LC group (*n* = 281)**	**HCC group (*n* = 280)**	***p*-value**	**1VS2**	**1VS3**	**2VS3**
Gender	Female	133	175	163	0.276	–	–	–
	Male	107	106	117				
Age		37 (24, 55)	35 (23, 61)	34 (22, 57)	0.982	–	–	–
AFP		2.99 (1.89, 4.56)	3.17 (2.06, 4.81)	3.11 (2.23, 4.53)	0.183	–	–	–
CEA		2.40 (1.44, 4.04)	2.69 (1.54, 3.87)	3.17 (1.78, 7.32)	<0.001	0.567	<0.001	<0.001
AST		31 (20, 57)	32 (23, 52)	33 (23, 49)	0.637	–	–	–
ALT		25 (15, 48)	30 (18, 54)	35 (20, 60)	<0.001	0.064	<0.001	0.113
TBIL		18.1 (11.5, 33.1)	19.0 (12.7, 28.9)	17.8 (13.1, 27.5)	0.752	–	–	–
DBIL		7.0 (4.0, 14.7)	6.3 (3.8, 10.9)	5.6 (4.0, 9.4)	0.008	0.181	0.006	0.668
TP		65.0 (57.0, 73.5)	63.8 (57.2, 71.15)	59.5 (54.8, 68.5)	<0.001	0.675	<0.001	<0.001
ALB		35.6 (30.0, 41.1)	32.8 (26.6, 37.8)	30.4 (25.6, 35.7)	<0.001	<0.001	<0.001	0.002
ALP		80 (59, 125)	83 (64, 114)	86 (66,116)	0.301	–	–	–
GGT		36 (19, 112)	42 (21, 91)	42 (21,91)	0.516	–	–	–

### The Expression Levels of ADA, AFU, and LAC Among Patients in the Three Groups

**Figure 1:**
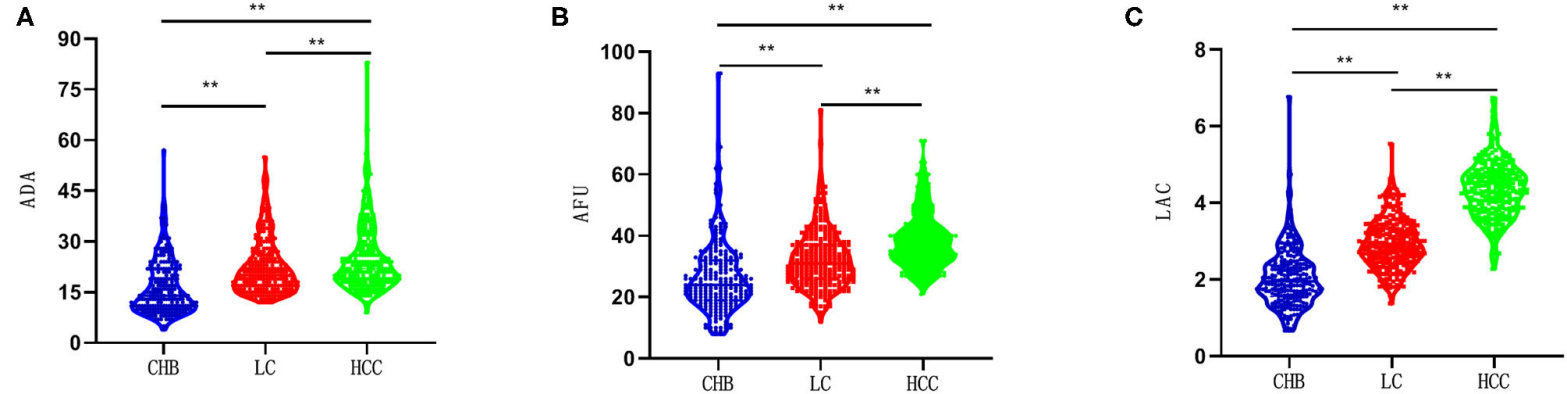
Comparison of the ADA, AFU, and LAC levels between patients in three groups. **p < 0.01. CHB, chronic hepatitis B; LC, related liver cirrhosis; HCC, hepatocellular carcinoma; ADA, adenosine deaminase; AFU, α-l-fucosidase; LAC, lactic acid. **(A)** the ADA levels in three groups; **(B)** the AFU levels in three groups; **(C)** the LAC levels in three groups.

The ADA levels of patients in the CHB, LC, and HCC groups were 14 U/L (11, 22), 20 U/L (17, 27), and 22 U/L (18, 29), respectively. The AFU levels of patients in the CHB, LC, and HCC groups were 24 U/L (19, 32), 31 U/L (26, 37), and 37 U/L (32, 43), respectively, while those of LAC in the three groups were 1.96 mmol/L (1.56, 2.42), 2.87 mmol/L (2.48, 3.33), and 4.34 mmol/L (3.84, 4.78), respectively. The levels of the three markers showed an increasing trend across groups, and the differences were statistically significant (all *p* < 0.05; Figures 1A–C).

### Diagnostic Performance of the Laboratory-Related Markers Among Patients in the CHB and LC Groups

The hepatitis group logit(P) (LC group = 1, HCC group = 0) was considered the dependent variable, and ADA (X1), AFU (X2), and LAC (X3) were considered the independent variables. Binary logistic regression analysis was performed to calculate the joint predictors of ADA, AFU, and LAC. The regression equation was logit(P) = −7.632 + 0.095X1 + 0.037X2 + 1.958X3, and the joint predictors were considered the three joint test indicators to analyze the results.

**Figure 2:**
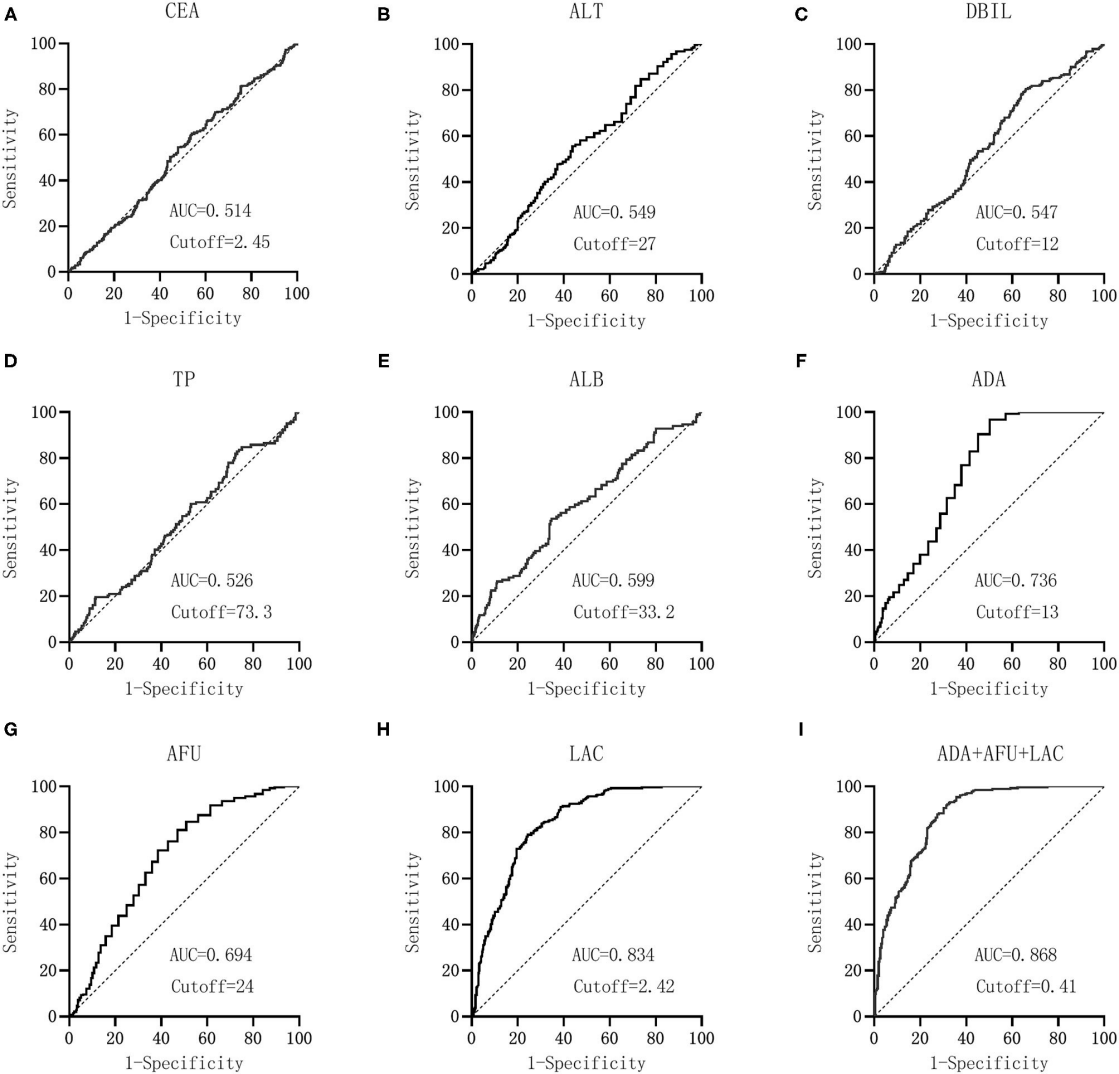
Diagnostic performance (ROC) of laboratory-related indicators in patients between CHB and LC groups. CEA, carcinoembryonic antigen; ALT, alanine aminotransferase; DBIL, direct bilirubin; TP, total protein; ALB, albumin; ADA, adenosine deaminase; AFU, α-l-fucosidase; LAC, lactic acid. GraphPad Prism software was used to the AUC of CEA **(A)**, ALT **(B)**, DBIL **(C)**, TP **(D)**, ALB **(E)**, ADA **(F)**, AFU **(G)**, LAC **(H)**, and ADA+AFU+LAC **(I)**.

GraphPad Prism software was used to construct the ROC curve of each index and combined test, as shown in Figures 2A–I. The AUC of CEA was 0.514 when the cutoff was 2.45 mg/L, and the sensitivity, specificity, NPV, and PPV were 54.45, 52.08, 49.4, and 57.1%, respectively. The AUC of ALT was 0.549 when the cutoff was 27 U/L, and the sensitivity, specificity, NPV, and PPV were 55.52, 55.83, 51.7, and 59.5%, respectively. Similarly, the AUC of DBIL was 0.547 when the cutoff was 12 μmol/L, and the sensitivity and specificity were 80.43 and 34.17%, respectively. The NPV and PPV of DBIL were 59.9 and 58.9%, respectively. The AUC of TP was 0.526 when the cutoff was 73.3 g/L, and the sensitivity, specificity, NPV, and PPV were 82.92, 26.67, 57.1, and 57.0%, respectively. The AUC of ALB was 0.599 when the cutoff was 33.2 g/L, and the sensitivity, specificity, NPV, and PPV were 53.74, 65.00, 54.5, and 64.3%, respectively. The AUC values of ADA, AFU, and LAC were 0.736, 0.694, and 0.834, respectively, when the cutoff values were 13 U/L, 24 U/L, and 2.42 mmol/L, respectively. The sensitivity, specificity, NPV, and PPV of ADA were 96.80, 46.67, 92.6, and 68.0%, respectively, whereas those of AFU were 81.14, 51.67, 70.1, and 66.3%, respectively. The sensitivity, specificity, NPV, and PPV of LAC were 79.00, 75.42, 75.4, and 79.0%, respectively. The AUC of the combined detection of ADA+AFU+LAC was 0.868 when the cutoff was 0.41. The sensitivity, specificity, NPV, and PPV of the combined detection were 91.81, 68.75, 87.8, and 77.5%, respectively. See Table 2.

**Table 2:** Comparative analysis of the results of laboratory-related indicators in CHB and LC groups.

**Indicators**	**Youden index**	**Cutoff value**	**AUC**	**Sensitivity**	**Specificity**	**AUC 95% CI**	**PPV (%)**	**NPV (%)**
CEA	0.065	2.45	0.514	54.45	52.08	0.470–0.558	57.1	49.4
ALT	0.114	27	0.549	55.52	55.83	0.505–0.592	59.5	51.7
DBIL	0.146	12	0.547	80.43	34.17	0.504–0.591	58.9	59.9
TP	0.096	73.3	0.526	82.92	26.67	0.482–0.569	57.0	57.1
ALB	0.187	33.2	0.599	53.74	65.00	0.556–0.641	64.3	54.5
ADA	0.435	13	0.736	96.80	46.67	0.696–0.773	68.0	92.6
AFU	0.328	24	0.694	81.14	51.67	0.653–0.733	66.3	70.1
LAC	0.544	2.42	0.834	79.00	75.42	0.799–0.865	79.0	75.4
ADA+AFU+LAC	0.606	0.41	0.868	91.81	68.75	0.836–0.896	77.5	87.8

From the data in Table 2, it could be concluded that the AUC values of ADA, AFU, LAC, and ADA+AFU+LAC were higher and that the diagnostic performance was superior. MedCalc software was used to compare the AUC values of ADA, AFU, LAC, and ADA+AFU+LAC. There was no statistically significant difference between the AUC values of ADA and AFU (*p* > 0.05). The AUC of LAC was greater than those of ADA and AFU, whereas the value of the combined detection was greater than those of ADA, AFU, and LAC alone, and the difference was statistically significant (*p* < 0.05; Table 3).

**Table 3:** Comparison of the AUC of ADA, AFU, LAC, and ADA+AFU+LAC in CHB and LC groups.

**Detection indicators**	***Z*-value**	***p*-value**
Combined test and ADA	5.963	<0.001
Combined test and AFU	7.180	<0.001
Combined test and LAC	2.814	0.005
ADA and AFU	1.497	0.135
ADA and LAC	3.279	0.001
AFU and LAC	4.725	<0.001

### Diagnostic Performance of the Laboratory-Related Markers Among Patients in the LC and HCC Groups

The hepatitis group logit(P) (HCC group = 1, LC group = 0) was considered the dependent variable, and ADA (X1), AFU (X2), and LAC (X3) were considered the independent variables. Binary logistic regression analysis was performed to calculate the joint predictors of ADA, AFU, and LAC. The regression equation was logit(P) = −7.632 + 0.095X1 + 0.037X2 + 1.958X3, and the joint predictors were used as the three joint test indicators to analyze the results.

**Figure 3:**
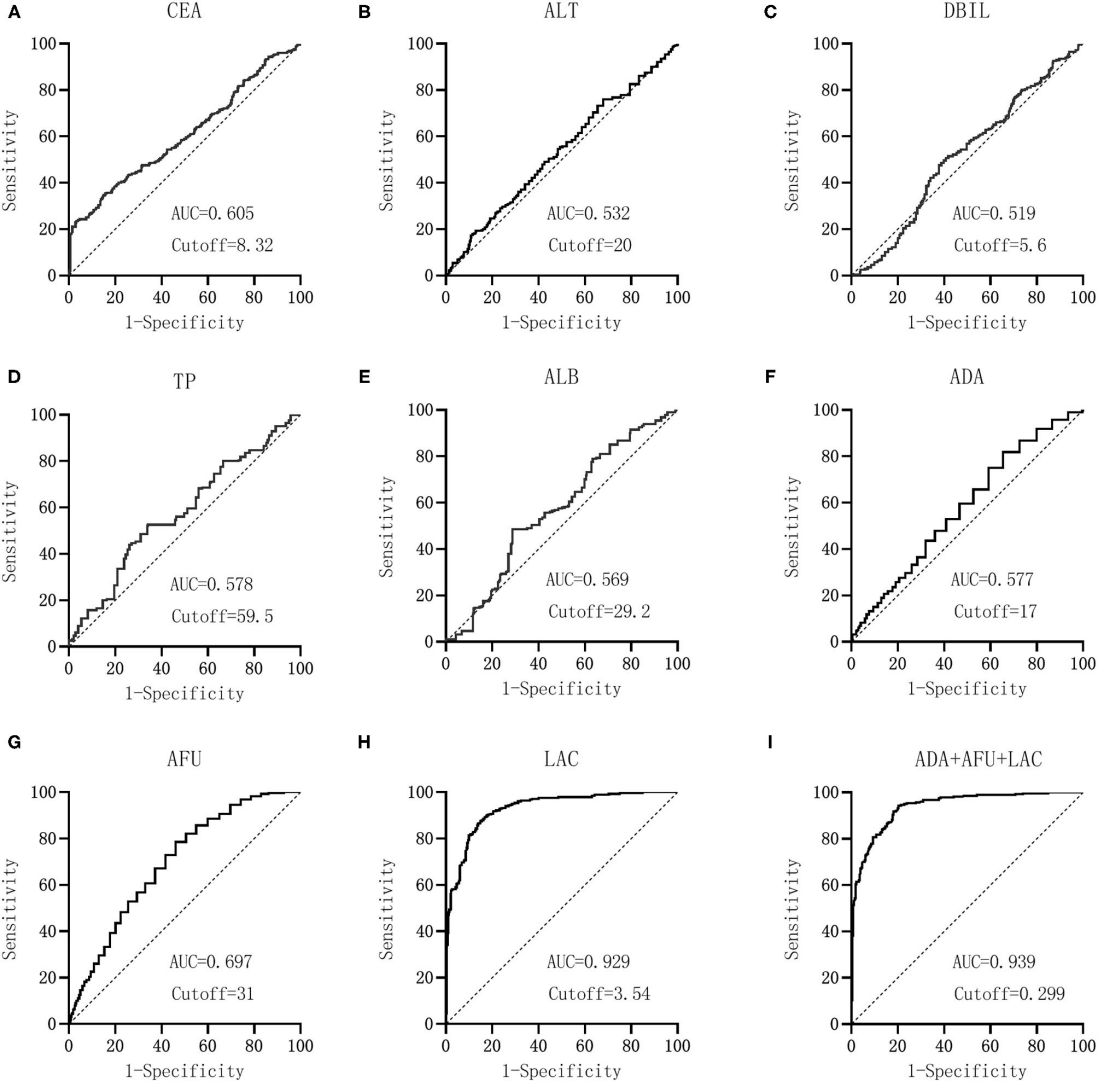
Diagnostic performance (ROC) of laboratory-relevant indicators in patients between CHB and LC groups. CEA, carcinoembryonic antigen; ALT, alanine aminotransferase; DBIL, direct bilirubin; TP, total protein; ALB, albumin; ADA, adenosine deaminase; AFU, α-l-fucosidase; LAC, lactic acid. GraphPad Prism software was used to the AUC of CEA **(A)**, ALT **(B)**, DBIL **(C)**, TP **(D)**, ALB **(E)**, ADA **(F)**, AFU **(G)**, LAC **(H)**, and ADA+AFU+LAC **(I)**. Diagnostic performance (ROC) of laboratory relevant indicators in patients between HCC and LC groups.

GraphPad Prism software was used to construct the ROC curve of each index and combined test, as shown in Figures 3A–I. The AUC of CEA was 0.605 when the cutoff was 8.32 mg/L, and the sensitivity, specificity, NPV, and PPV were 23.57, 96.80, 56.0, and 88.0%, respectively. The AUC of ALT was 0.532 when the cutoff was 20 U/L, and the sensitivity, specificity, NPV, and PPV were 73.21, 33.81, 55.9, and 52.4%, respectively. The AUC of DBIL was 0.519 when the cutoff was 5.6 μmol/L, and the sensitivity and specificity were 50.71 and 59.07%, respectively. The NPV and PPV of DBIL were 54.6 and 55.3%, respectively. The AUC of TP was 0.578 when the cutoff was 59.5 g/L, and the sensitivity, specificity, NPV, and PPV were 52.14, 66.19, 58.1, and 60.6%, respectively. The AUC of ALB was 0.569 when the cutoff was 29.2 g/L, and the sensitivity, specificity, NPV, and PPV were 48.57, 71.17, 58.1, and 62.7%, respectively. The AUC values of ADA, AFU, and LAC were 0.577, 0.697, and 0.929, respectively, when the cutoff values were 17 U/L, 31 U/L, and 3.54 mmol/L, respectively. The sensitivity, specificity, NPV, and PPV of ADA were 81.19, 31.67, 63.6, and 54.4%, respectively, whereas those of AFU were 78.57, 51.60, 70.7, and 61.8%, respectively. The sensitivity, specificity, NPV, and PPV of LAC were 87.50, 85.05, 87.2, and 85.4%, respectively. The AUC of the combined detection of ADA+AFU+LAC was 0.939 when the cutoff was 0.299. The sensitivity, specificity, NPV, and PPV of the combined detection were 94.29, 79.72, 93.3, and 82.2%, respectively. See Supplementary Table 1.

From the data in Table 2, it could be concluded that the AUC values of ADA, AFU, LAC, and ADA+AFU+LAC were higher and that the diagnostic performance was superior. MedCalc software was used to compare the AUC values of ADA, AFU, LAC, and ADA+AFU+LAC. The AUC of LAC was greater than that of AFU, whereas the value of AFU was greater than that of ADA. Furthermore, the AUC of the combined detection was greater than that of ADA, AFU, and LAC alone, and the difference was statistically significant (*p* < 0.05; Supplementary Table 2).

### Risk Assessment of ADA, AFU, and LAC in Predicting LC

**Figure 4:**
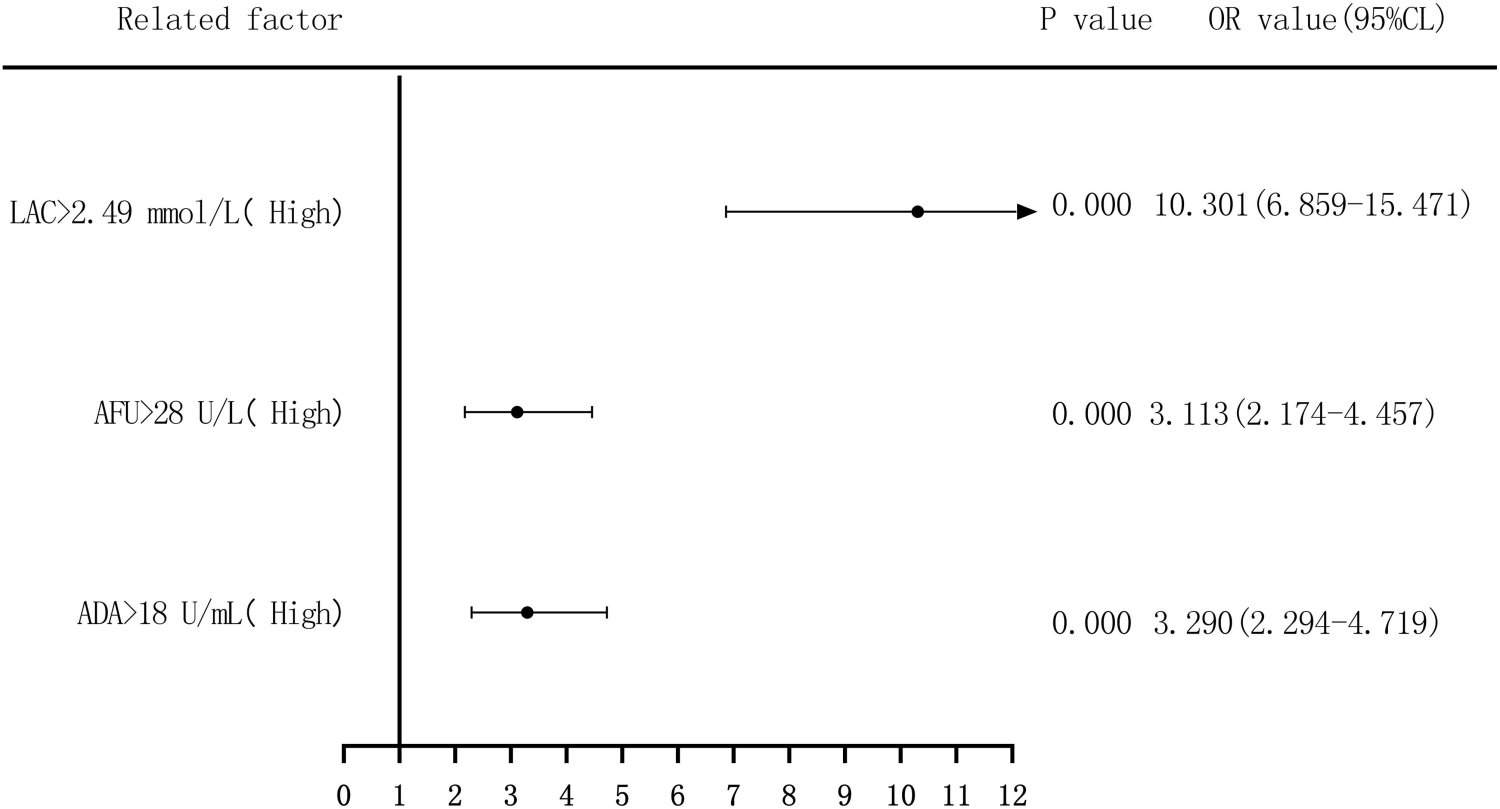
Forest plots of univariate logistic regression analysis of ADA, AFU, LAC and patients (two-group classification) with liver cirrhosis.

**Figure 5:**
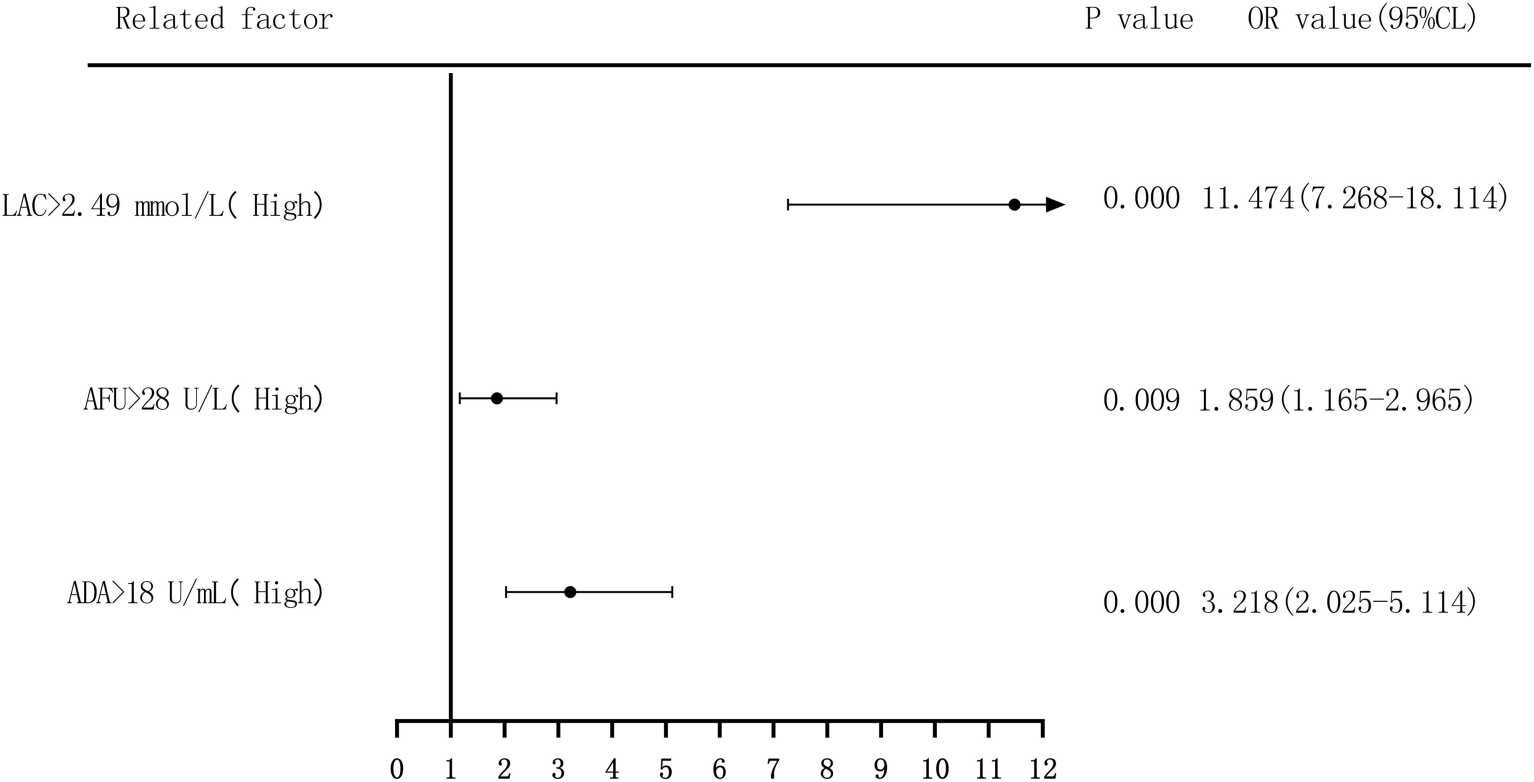
Forest plots of multivariate logistic regression analysis of ADA, AFU, LAC, and patients (two-group classification) with liver cirrhosis (Multi-factor adjustment includes variables ALB, ALT, ADA, AFU, LAC).

Binary logistic regression analysis was performed to evaluate the risk of ADA, AFU, and LAC levels in patients with LC, with the median and quartiles as the cutoff points (two-group and four-group classifications, respectively). First, based on the median value of ADA (18 U/L), AFU (28 U/L), and LAC (2.49 mmol/L), we divided patients into low-level and high-level groups. Regarding the risk of developing LC, compared to the patients in the low-level group, patients with high ADA levels had an odds ratio (OR) value of 3.290 (95% CI, 2.294–4.719; *p* < 0.05), and the adjusted OR was 3.218 (95% CI, 2.025–5.114; *p* < 0.05). Similarly, compared to the patients in the low-level group, patients with high AFU levels had an OR value of 3.113 (95% CI, 2.174–4.457; *p* < 0.05), and the adjusted OR was 1.859 (95% CI, 1.165–2.965; *p* < 0.05). The OR for the risk of developing LC in patients with high LAC levels was 10.301 (6.859–15.471; *p* < 0.05) compared to those with low levels, and the adjusted OR was 11.474 (95% CI, 7.268–18.114; *p* < 0.05; Figures 4, 5).

**Figure 6:**
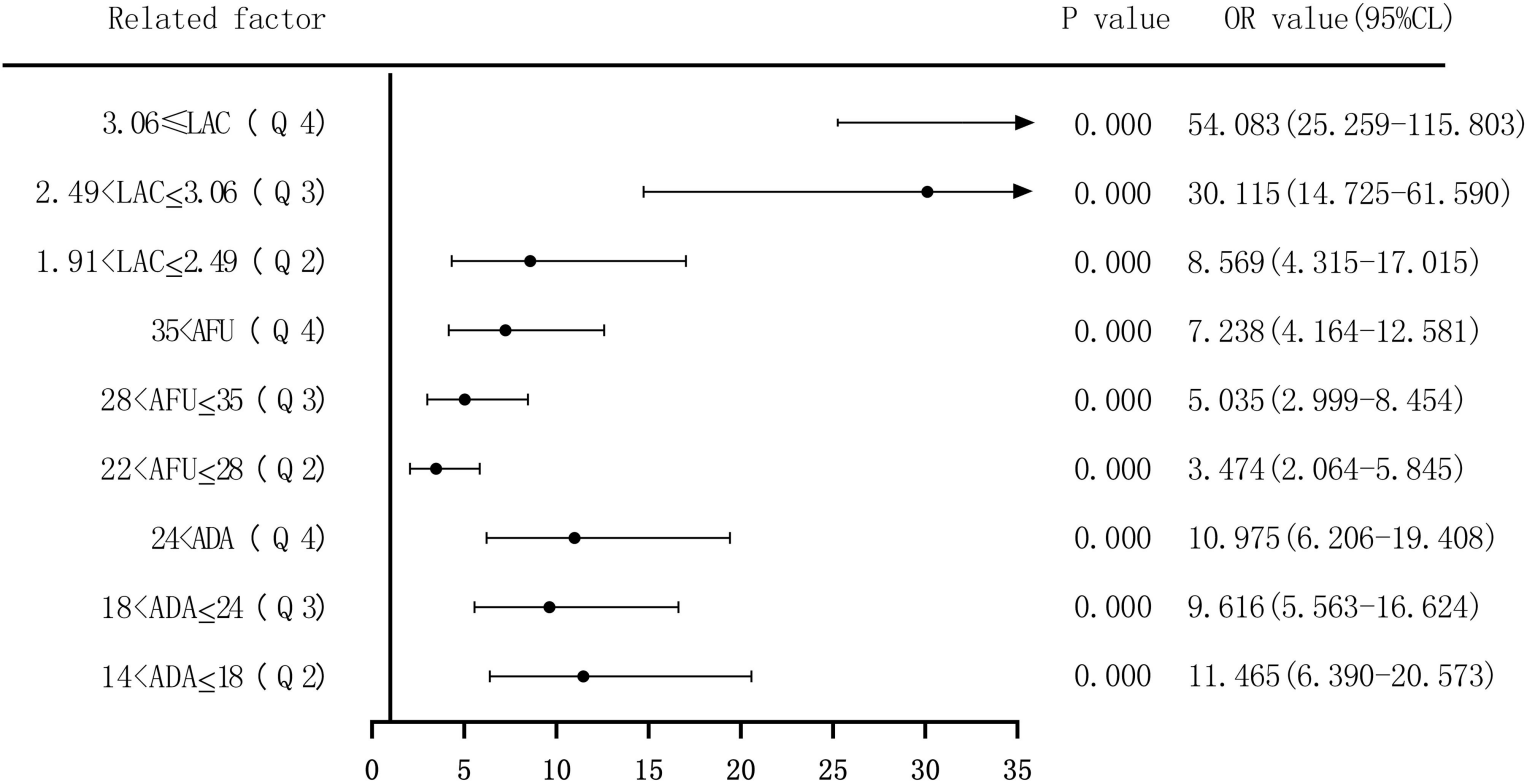
Forest plots of univariate logistic regression analysis of ADA, AFU, LAC, and liver cirrhosis patients (four-group classification).

**Figure 7:**
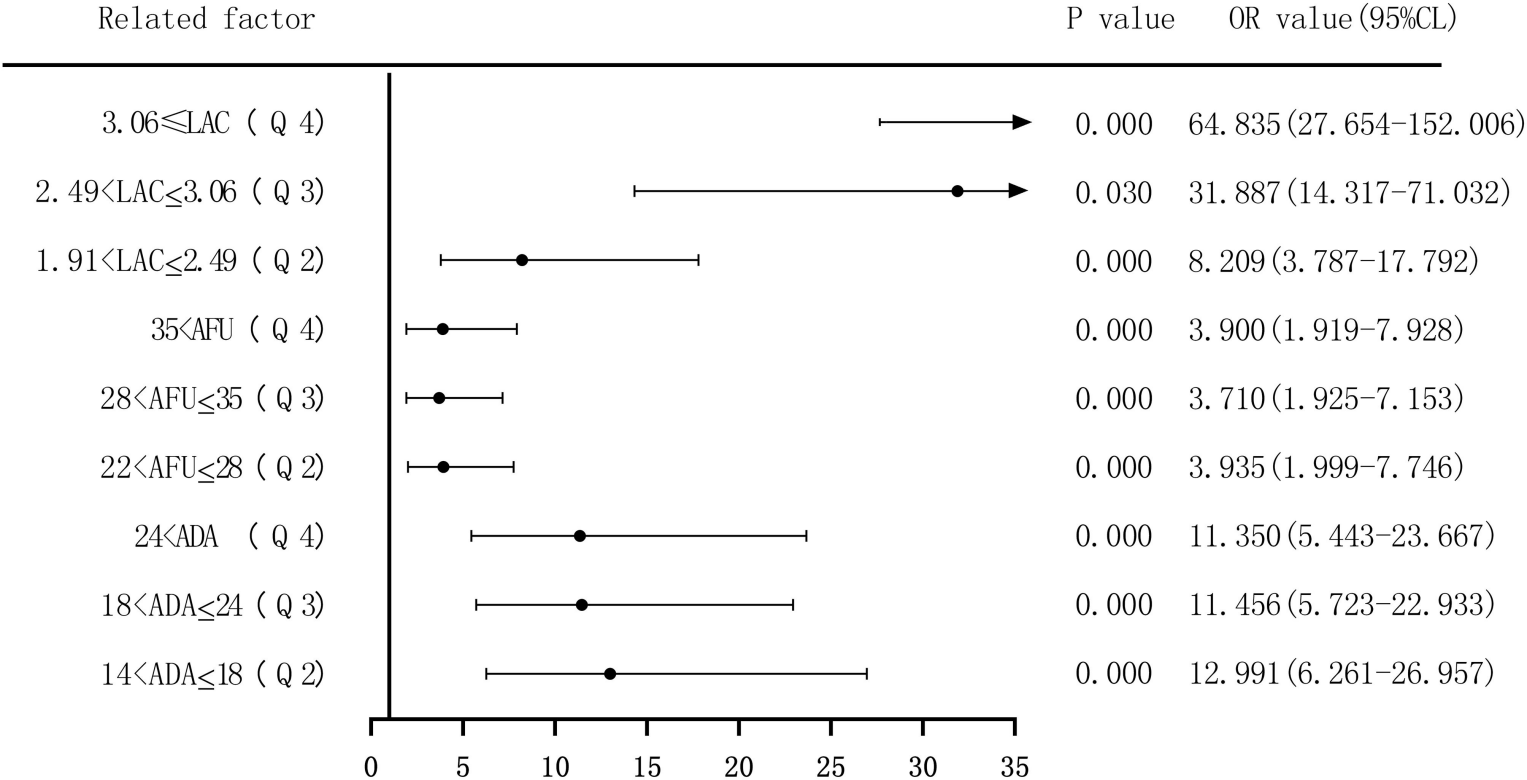
Forest plots of multivariate logistic regression analysis of ADA, AFU, LAC, and liver cirrhosis patients (four-group classification) (Multi-factor adjustment includes variables ALB, ALT, ADA, AFU, LAC).

Second, based on the quartile values of ADA (Q1 ≤ 14; 14 < Q2 ≤ 18; 18 < Q3 ≤ 24; and 24 < Q4), AFU (Q1 ≤ 22; 22 < Q2 ≤ 28; 28 < Q3 ≤ 35; and 35 < Q4), and LAC (Q1 ≤ 1.91; 1.91 < Q2 ≤ 2.49; 2.49 < Q3 ≤ 3.055; and 3.055 < Q4), the patients were divided into Q1 (lowest), Q2, Q3, and Q4 groups from low to high levels. Compared to that of the group with the lowest ADA level (Q1), the OR values for the risk of developing LC in the Q2, Q3, and Q4 groups were 11.465 (6.390–20.573), 9.616 (5.563–16.624), and 10.975 (6.206–19.408), respectively, and the adjusted OR values were 12.991 (6.261–26.957), 11.456 (5.723–22.933), and 11.350 (5.443–23.667), respectively. Similarly, based on AFU levels, compared to that of the Q1 group, the OR values for the risk of developing LC in the Q2, Q3, and Q4 groups were 3.474 (2.064–5.845), 5.035 (2.999–8.454), and 7.238 (4.164–12.581), respectively, and the adjusted OR values were 3.935 (1.999–7.746), 3.710 (1.925–7.153), and 3.900 (1.919–7.928), respectively. The OR values for the risk of developing LC based on LAC levels in the Q2, Q3, and Q4 groups were 8.569 (4.315–17.015), 30.115 (14.725–61.590), and 54.083 (25.259–115.803), respectively, and the adjusted OR values were 8.209 (3.787–17.792), 31.887 (14.314–71.032), and 64.835 (27.654–152.006), respectively, compared to that in the Q1 group. See Figures 6, 7.

### Risk Assessment of ADA, AFU, and LAC in Predicting HCC

**Figure 8:**
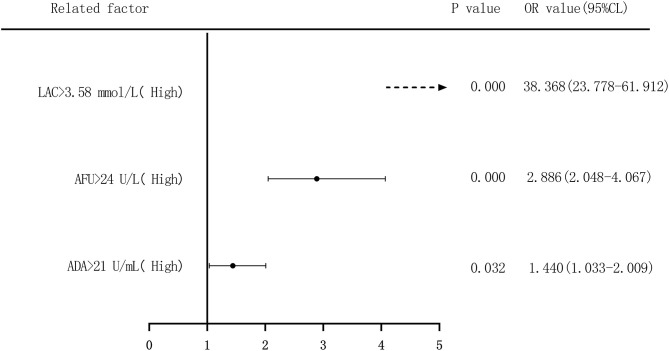
Forest plots of univariate logistic regression analysis of ADA, AFU, LAC, and HCC patients (Note: Dotted line = OR value exceeds the range shown in the figure).

**Figure 9:**
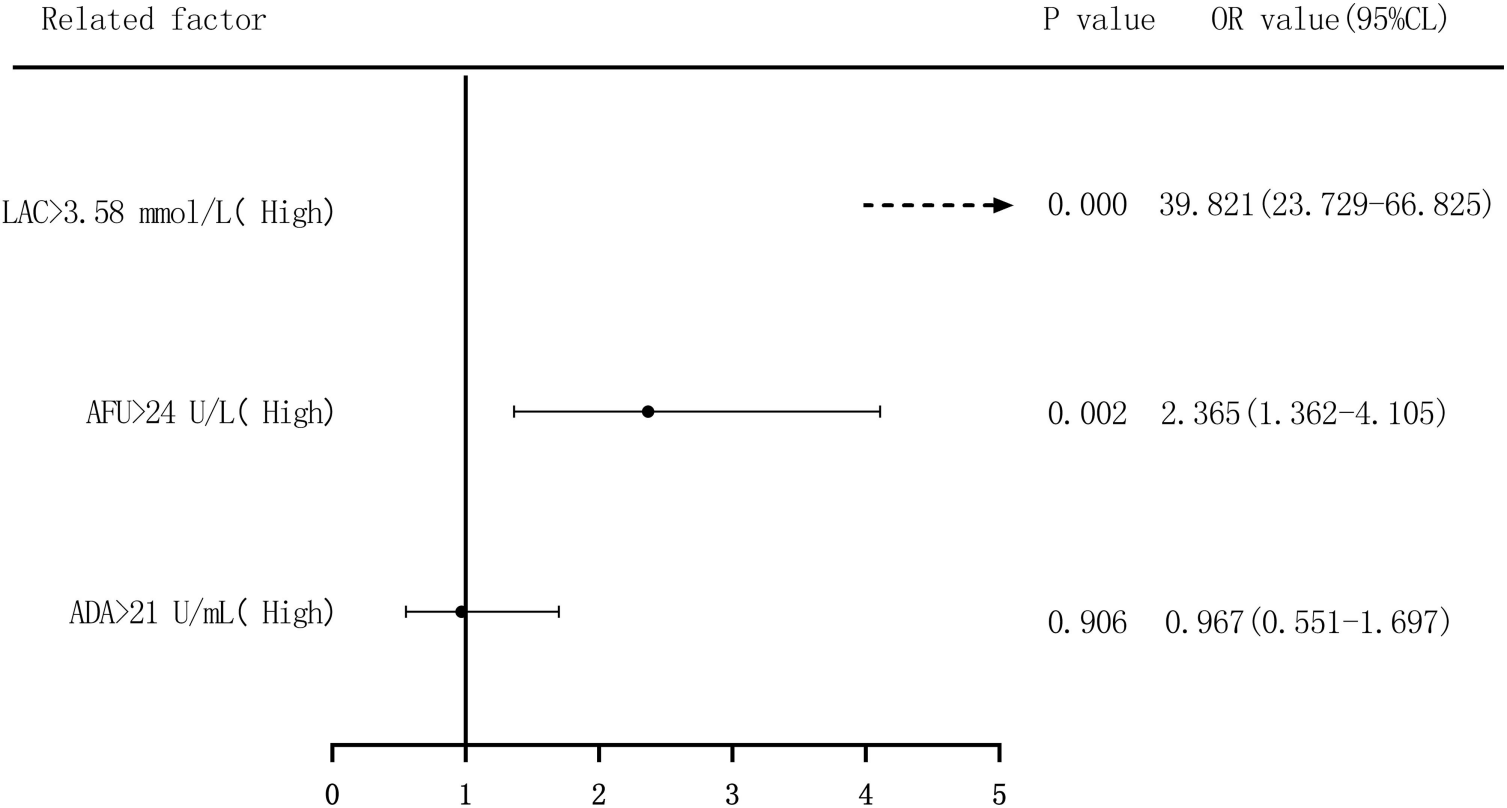
Forest plots of multivariate logistic regression analysis of ADA, AFU, LAC, and patients (two-group classification) with hepatocellular carcinoma (Multi-factor adjustment includes variables CEA, TP, ALB, ADA, AFU, LAC; dotted line = OR value exceeds the range shown in the figure).

We performed binary logistic regression analysis to evaluate the risk of ADA, AFU, and LAC levels in patients with HCC, considering the median and quartiles as the cutoff points (two-group and four-group classifications, respectively). First, based on the median value of ADA (21 U/L), AFU (34 U/L), and LAC (3.58 mmol/L), patients were divided into low-level and high-level groups. Regarding the risk of developing HCC, compared to the patients in the low-level group, patients with high ADA levels had an OR value of 1.440 (95% CI, 1.033–2.009; *p* < 0.05), and the adjusted OR was 0.967 (95% CI, 0.551–1.697; *p* > 0.05). Similarly, compared to the patients in the low-level group, patients with high AFU levels had an OR value of 2.886 (95% CI, 2.048–4.067; *p* < 0.05), and the adjusted OR was 2.365 (95% CI, 1.362–4.105; *p* < 0.05). The OR for the risk of developing HCC in patients with high LAC levels was 38.368 (23.778–61.912; *p* < 0.05) compared to those with low levels, and the adjusted OR was 39.821 (95% CI, 23.729–66.825; *p* < 0.05; Figures 8, 9).

**Figure 10:**
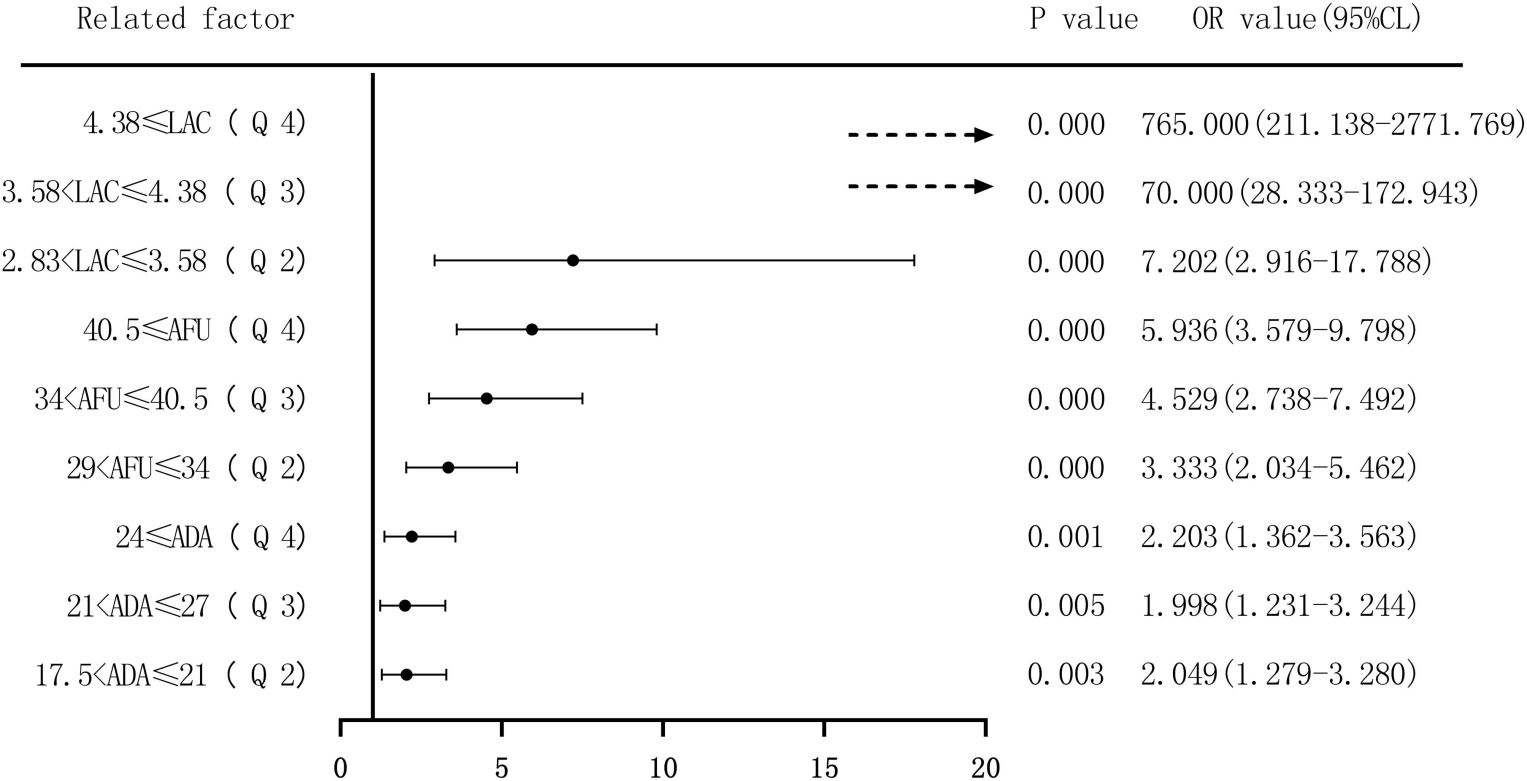
Forest plots of univariate logistic regression analysis of ADA, AFU, LAC, and hepatocellular carcinoma patients (four-group classification) (Dotted line = OR value exceeds the range shown in the figure).

**Figure 11:**
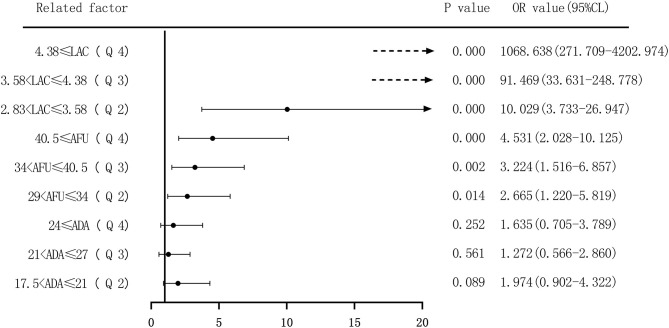
Forest plots of multivariate logistic regression analysis of ADA, AFU, LAC, and hepatocellular carcinoma patients (four-group classification) (Multi-factor adjustment includes variables CEA, TP, ALB, ADA, AFU, LAC; dotted line = OR value exceeds the range shown in the figure).

Second, based on the quartile values of ADA (Q1 ≤ 17.5; 17.5 < Q2 ≤ 21; 21 < Q3 ≤ 27; and 27 < Q4), AFU (Q1 ≤ 29; 29 < Q2 ≤ 34; 34 < Q3 ≤ 40.5; and 40.5 < Q4), and LAC (Q1 ≤ 2.83; 2.83 < Q2 ≤ 3.58; 3.58 < Q3 ≤ 4.38; and 4.38 < Q4), we divided the patients into Q1 (lowest), Q2, Q3, and Q4 groups from low to high levels. Compared to that of the lowest ADA level group (Q1), the OR values for the risk of developing HCC in the Q2, Q3, and Q4 groups were 2.049 (1.279–3.280), 1.998 (1.231–3.244), and 2.203 (1.362–3.563), respectively, and the adjusted OR values were 1.974 (0.902–4.322), 1.272 (0.566–2.86), and 1.635 (0.705–3.789), respectively. Similarly, based on AFU levels, compared to that in the Q1 group, the OR values for the risk of developing HCC in the Q2, Q3, and Q4 groups were 3.333 (2.034–5.462), 4.529 (2.738–7.492), and 5.936 (3.597–9.798), respectively, and the adjusted OR values were 2.665 (1.220–5.819), 3.224 (1.516–6.857), and 4.531 (2.028–10.125), respectively. The OR values for the risk of developing HCC based on LAC levels in the Q2, Q3, and Q4 groups were 7.202 (2.916–17.788), 70.000 (28.333–172.943), and 765.000 (211.138–2771.769), respectively, and the adjusted OR values were 10.029 (3.733–26.947), 91.469 (33.631–248.778), and 1068.638 (271.709–4202.974) compared to the Q1 group. See Figures 10, 11.

## Discussion

HBV infection poses a major health threat to humans. Infected patients can develop CHB and gradually LC or HCC (15, 16). LC is also a major risk factor for HCC. Therefore, screening and early diagnosis of LC and HCC are significant for patients with CHB and subsequent cirrhosis.

This study mainly discussed the diagnostic value of ADA, AFU, and LAC levels in patients with early-stage CHB, LC, and HCC. The results revealed that ADA levels in patients with CHB, LC, and HCC were 14 U/L (11, 22), 20 U/L (17, 27), and 22 U/L (18, 29), respectively, which showed an increasing trend, and the difference was statistically significant. Similarly, the levels of AFU in patients with CHB, LC, and HCC were 24 U/L (19, 32), 31 U/L (26, 37), and 37 U/L (32, 43), respectively, while those of LAC were 1.96 mmol/L (1.56, 2.42), 2.87 mmol/L (2.48, 3.33), and 4.34 mmol/L (3.84, 4.78), respectively. The values showed an increasing trend, and the differences were statistically significant. The results of our study are in agreement with those reported by Yu et al., who observed a higher expression of ADA in patients with HCC (17). In addition, the present study demonstrated that the level of ADA was higher in patients with LC. The LAC level in the blood can be considered to measure the oxygen metabolism and status of tissue perfusion in the human body. Liver failure caused by liver function metabolism has been shown to increase the level of LAC (18). This observation is consistent with the results of the present study confirming the increase in LAC levels with the progression of liver disease.

The ROC curve of each index and combined test produced by the GraphPad Prism software revealed that the AUC values of ADA, AFU, LAC, and ADA+AFU+LAC in the CHB and LC groups were 0.736, 0.694, 0.834, and 0.868, respectively, and that the diagnostic performance was superior. Comparison of the AUC values of ADA, AFU, LAC, and ADA+AFU+LAC revealed that the difference between ADA and AFU was not statistically significant. The AUC of LAC was greater than that of ADA and AFU. The AUC of the combined detection was greater than that of ADA, AFU, and LAC alone, and the difference was statistically significant. The results indicated that the diagnostic performance of LAC was superior to that of ADA and AFU; however, there was no advantage between ADA and AFU. Furthermore, the combined detection of ADA+AFU+LAC was superior to single detection for the diagnosis of LC. The AUC values of ADA, AFU, LAC, and ADA+AFU+LAC in the LC and HCC groups were 0.577, 0.697, 0.929, and 0.939, respectively, and the diagnostic performance was superior. The results are consistent with the value of AFU reported in the study by Xing et al. for the diagnosis of early HCC (19). Analysis of the AUC values of ADA, AFU, LAC, and ADA+AFU+LAC revealed that the AUC of LAC was greater than that of AFU. Moreover, the value of AFU was greater than that of ADA. The AUC of the combined detection was greater than that of ADA, AFU, and LAC alone, and there was a statistically significant difference, indicating that the three markers showed an upward trend in the early diagnosis of HCC. In addition, the combined detection of ADA+AFU+LAC was superior to single detection for the early diagnosis of HCC.

The risk of ADA, AFU, and LAC levels in patients with early LC and HCC was assessed by binary logistic regression analysis, and the median and quartiles were considered the cutoff points (two-group and four-group classifications, respectively). The risk assessment in patients with LC demonstrated that when the median was considered the cutoff point in the two-group classification, the adjusted OR values of ADA, AFU, and LAC for the risk of developing LC among patients in the high-level group compared to those in the low-level group were 3.218, 1.859, and 11.474, respectively. When quartiles were considered the cutoff point in the four-group classification, the adjusted OR values of ADA, AFU, and LAC were higher than those in the lowest-level group (Q1), and the difference was statistically significant. The results show that ADA, AFU, and LAC can be considered risk predictors of LC. The risk assessment in patients with HCC also showed that when the median was considered the cutoff point in the two-group classification, the adjusted OR values of ADA, AFU, and LAC for the risk of developing LC among patients in the high-level group compared to those in the low-level group were 0.967, 2.365, and 38.368, respectively. When quartiles were considered the cutoff point for the four-group classification, the adjusted OR values of AFU and LAC compared to the lowest-level group (Q1) were higher than that of the Q1 group, and the difference was statistically significant. There was no statistically significant difference between the adjusted ADA level and the lowest-level group (Q1). The results reveal that AFU and LAC, but not ADA, can be considered early risk predictors of HCC.

In summary, detection of ADA, AFU, and LAC has good value in the early diagnosis of LC and HCC. The combined detection of ADA+AFU+LAC is superior to single detection for the early diagnosis of LC and HCC. ADA, AFU, and LAC can be considered risk predictors of LC. Furthermore, AFU and LAC can be considered early risk predictors of HCC; however, the predictive ability of ADA is insufficient. It is worth noting that due to the small sample size and the failure to consider factors such as the use of drugs during the treatment of patients, further research is required with a larger sample size and a prospective study design to investigate the value of various markers in the early diagnosis of LC and HCC.

## Data Availability Statement

The original contributions presented in the study are included in the article/Supplementary Material, further inquiries can be directed to the corresponding author/s.

## Ethics Statement

The studies involving human participants were reviewed and approved by the Ethics Committees of Jiaozuo Fifth People's Hospital (Approval No. 20150762). Written informed consent to participate in this study was provided by the participants' legal guardian/next of kin.

## Author Contributions

WZ, ZC, and CX contributed to study concept and design, acquisition of the data, analysis and interpretation of the data, and drafting of the manuscript. YZ and LW contributed to statistical analysis. JZ contributed to sample collections. SX, JT, and ZP contributed to study concept and design, study supervision, and critical revision of the manuscript. All authors have read and approved the manuscript.

## Conflict of Interest

The authors declare that the research was conducted in the absence of any commercial or financial relationships that could be construed as a potential conflict of interest.

## Publisher's Note

All claims expressed in this article are solely those of the authors and do not necessarily represent those of their affiliated organizations, or those of the publisher, the editors and the reviewers. Any product that may be evaluated in this article, or claim that may be made by its manufacturer, is not guaranteed or endorsed by the publisher.

## Abbreviations

ADA: adenosine deaminase
AFU: α-l-fucosidase
LAC: lactic acid
CHB: chronic hepatitis B
LC: liver cirrhosis
HCC: hepatocellular carcinoma
NPV: negative predictive value
PPV: positive predictive value
AUC: area under the ROC curve.
