# Supplementary material for: The Applicability of ADA, AFU, and LAC in the Early Diagnosis and Disease Risk Assessment of Hepatitis B-Associated Liver Cirrhosis and Hepatocellular Carcinoma
Source: Front Med (Lausanne). 2021 Sep 7;8:740029. doi: 10.3389/fmed.2021.740029 (PMC8453082; doi:10.3389/fmed.2021.740029)
Supplement: Supplementary file 1 [file Data_Sheet_1.docx]

**Table S1 Comparative analysis of the results of laboratory-related indicators of patients in CHB and LB groups**

| Indicators | Yorden index | Cutoff value | AUC | Sensitivity | Specificity | AUC 95%CI | PPV（%） | NPV（%） |
| --- | --- | --- | --- | --- | --- | --- | --- | --- |
| CEA | 0.204 | 8.32 | 0.605 | 23.57 | 96.80 | 0.563-0.646 | 88.0 | 56.0 |
| ALT | 0.070 | 20 | 0.532 | 73.21 | 33.81 | 0.490-0.574 | 52.4 | 55.9 |
| DBIL | 0.098 | 5.6 | 0.519 | 50.71 | 59.07 | 0.477-0.561 | 55.3 | 54.6 |
| TP | 0.183 | 59.5 | 0.578 | 52.14 | 66.19 | 0.536-0.619 | 60.6 | 58.1 |
| ALB | 0.198 | 29.2 | 0.569 | 48.57 | 71.17 | 0.527-0.611 | 62.7 | 58.1 |
| ADA | 0.135 | 17 | 0.577 | 81.19 | 31.67 | 0.535-0.618 | 54.4 | 63.6 |
| AFU | 0.302 | 31 | 0.697 | 78.57 | 51.60 | 0.657-0.735 | 61.8 | 70.7 |
| LAC | 0.726 | 3.54 | 0.929 | 87.50 | 85.05 | 0.905-0.949 | 85.4 | 87.2 |
| ADA+AFU+LAC | 0.740 | 0.299 | 0.939 | 94.29 | 79.72 | 0.916-0.957 | 82.2 | 93.3 |

**Table S2 Comparison of the AUC detected by ADA, AFU, LAC and ADA+AFU+LAC in LC and HCC groups**

| Detection indicators | *Z* value | *P* value |
| --- | --- | --- |
| Combined test and ADA | 0.362 | ＜0.001 |
| Combined test and AFU | 0.241 | ＜0.001 |
| Combined test and LAC | 2.182 | 0.029 |
| ADA and AFU | 4.442 | ＜0.001 |
| ADA and LAC | 13.144 | ＜0.001 |
| AFU and LAC | 9.487 | ＜0.001 |
